# Comparison of Minimally Invasive Monitoring Methods and Live Trapping in Mammals

**DOI:** 10.3390/genes12121949

**Published:** 2021-12-03

**Authors:** Andrea Miranda Paez, Mekala Sundaram, Janna R. Willoughby

**Affiliations:** 1 School of Forestry and Wildlife Sciences, Auburn University, Auburn, AL 36849, USA; jwilloughby@auburn.edu; 2 Odum School of Ecology, Center for Ecology of Infectious Diseases, University of Georgia, Athens, GA 30602, USA; mekala.sundaram@gmail.com

**Keywords:** camera trap, census, density, genetic, hair, live traps, population, scat

## Abstract

The conservation and management of wildlife requires the accurate assessment of wildlife population sizes. However, there is a lack of synthesis of research that compares methods used to estimate population size in the wild. Using a meta-analysis approach, we compared the number of detected individuals in a study made using live trapping and less invasive approaches, such as camera trapping and genetic identification. We scanned 668 papers related to these methods and identified data for 44 populations (all focused on mammals) wherein at least two methods (live trapping, camera trapping, genetic identification) were used. We used these data to quantify the difference in number of individuals detected using trapping and less invasive methods using a regression and used the residuals from each regression to evaluate potential drivers of these trends. We found that both trapping and less invasive methods (camera traps and genetic analyses) produced similar estimates overall, but less invasive methods tended to detect more individuals compared to trapping efforts (mean = 3.17 more individuals). We also found that the method by which camera data are analyzed can significantly alter estimates of population size, such that the inclusion of spatial information was related to larger population size estimates. Finally, we compared counts of individuals made using camera traps and genetic data and found that estimates were similar but that genetic approaches identified more individuals on average (mean = 9.07 individuals). Overall, our data suggest that all of the methods used in the studies we reviewed detected similar numbers of individuals. As live trapping can be more costly than less invasive methods and can pose more risk to animal well-fare, we suggest minimally invasive methods are preferable for population monitoring when less-invasive methods can be deployed efficiently.

## 1. Introduction

Methods of monitoring wildlife populations focus on measures of density or abundance of populations, which allow for the evaluation of the dynamics of populations over time and in response to management strategies [1]. Population estimates can also support the evaluation of the viability of a population, population size, hunting limits, and examining impacts of changes in the environment and system [1]. However, when it comes to rare or difficult-to-catch species, especially species from small populations or those that occur at low densities, trapping or otherwise capturing individuals can be extremely time consuming. Although there are many methods for monitoring wildlife such as camera trapping or minimally invasive DNA analysis, all methods have their limitations [2]. Identifying methods that provide accurate estimates efficiently is essential for monitoring species and managing ecosystems.

One traditional method for obtaining data to assess population size or density is live trapping [3]. With live trapping, individuals of the target species in a study are trapped and released between each sampling session, creating capture histories of individuals and providing information about the individuals captured, such as overall health, sex, and even reproductive status [4,5]. However, this method can be extremely labor intensive and, as a result, cost restrictive and time consuming. Traps are often checked in 12-h intervals but, depending on the species of study, traps may be checked more often. For example, insectivore traps need to be checked much more frequently as their diet does not provide the energy to withstand long hours in a trap [5]. In addition, the potential to stress an animal and interfere with their daily activity is high, thereby limiting the quality of the data [3]. Although these effects can be limited by methodological choices cleared by animal ethics authorities [5], less stressful and more efficient monitoring methods are often desirable.

Since their development in the early 1980s, camera traps have been used to study population sizes especially for large carnivores with distinctive natural marks (e.g., *Panthera tigris*, *Panthera onca*, and *Lynx rufus*) [6]. Camera traps are noninvasive methods useful for species inventories, estimating population density, calculating home range, and monitoring population dynamics [7]. The advantages of using camera traps, compared to other methods, is that they have a relatively low cost and do not require physical or chemical animal restraint, avoiding capture stress [6,7]. Natural marks and fur patterns help identify individuals and establish capture history [8]. However, using camera traps is often restricted by the ability to identify individuals or species that do not have distinctive fur or marking patterns [9]. Likewise, using camera traps to estimate abundance alone can be a challenge when the movement area of animals is not known, requiring costly telemetry to support population size estimates [10].

One alternative to camera traps that is often less invasive than trapping is the use of genetic-based methods for individual identification [11]. With genetic sampling, capture histories can be constructed through the captured genotypes from samples of tissue, scat, or hair [4]. Genetic data captured can also reveal other patterns important to long-term population stability such as inbreeding rates, genetic diversity, population structure, and patterns of gene flow [12]. While scat samples can be collected opportunistically, hair snagging devices can also be deployed with lures or bait [13]. As samples like scat and hair are often easier to obtain compared to the efforts related to trapping, reduced field costs can help to make this method more cost-efficient, enabling additional collection and larger sample sizes [4]. However, there are drawbacks to DNA-based techniques. When samples remain uncollected immediately after being deposited, the DNA will degrade. This can lead to genotyping errors such as false alleles and allelic dropout, inflating the number of unique individuals identified. Through repeated amplification of the genetic samples, these genotyping errors can be reduced, although the repeated genetic work comes with additional sample processing cost [4].

Minimally invasive sampling techniques are often preferred for endangered species because these methods generally pose less risk of injury or death compared to trapping or other approaches. Monitoring population densities using cameras or genetic analyses, especially in tandem, may provide insight into the trade-offs of capture methodologies. However, the overall pattern of detections that are made when using live trapping compared to those made using less invasive methods (i.e., those using camera traps or genetic data) across species is unknown. The primary objective of this study was to compile literature that compared individual detections using at least two common methods: live trapping, camera trapping, and genetic analysis. Using these data, we answered three questions: (1) “Does the number of individuals detected change when using live trapping methods compared to when less invasive methods (i.e., camera trapping and genetic analyses) are used?”; (2) “Do estimates from minimally invasive data collection methodologies have a similar sensitivity in identifying unique individuals?”; and (3) “What species and study-specific criteria are associated with differences in effectiveness of live traps, camera, and genetic data?”. Understanding how these methods differ in their ability to identify unique individuals will be important for targeting the correct methodologies for estimating population sizes in on-going management work.

## 2. Materials and Methods

**Figure 1:**
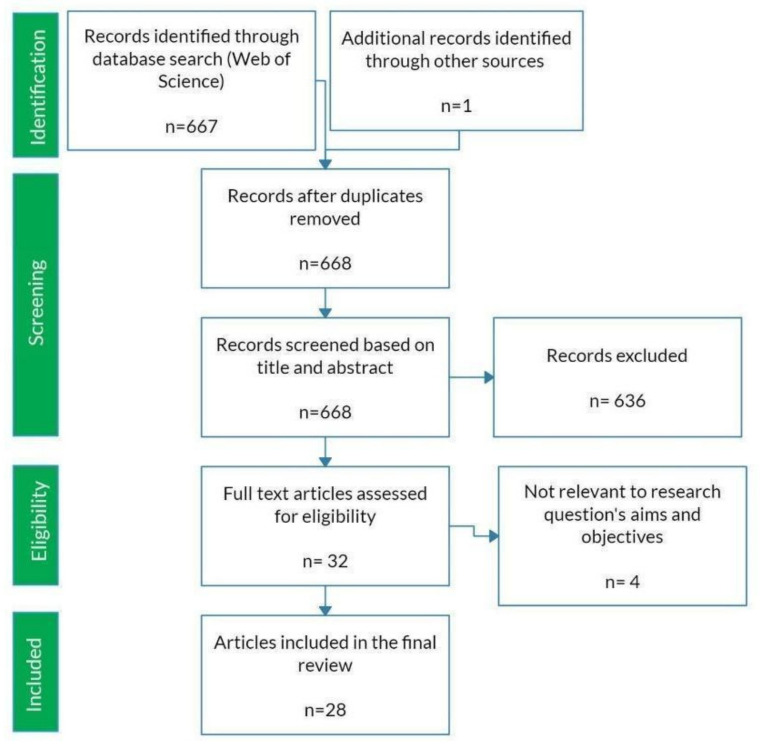
PRISMA diagram of our search protocol and results. Hundreds of papers were identified through database search using our three sets of keywords. Title and abstracts were then reviewed as the first screening process. Papers that contained method comparison between trapping and less invasive methods, as well as camera and genetic methods were retained. After screening, 32 full-text articles were reviewed for eligibility and 4 full texts were excluded because they did not contain comparisons of population size from at least two methods. In total, 28 full-text manuscripts were included in our data set.

We used a systematic literature search to identify existing data using the Web of Science. We conducted three searches using the following sets of keywords: (1) (hair OR scat OR gene*) AND (census OR density) AND (live trap*); (2) (camera trap* OR camera-trap* OR game camera* OR trail camera*) AND (census OR density) AND (live trap*); (3) (camera trap* OR camera-trap* OR game camera* OR trail camera*) AND (hair OR scat OR gene*) AND (census OR density). We included articles from 1900–present and our final list of papers included one non-peer-reviewed, preprint manuscript. From this set of papers, we identified studies that reported the number of individuals they detected in the focal species using at least two of the following methods to identify individuals: live traps, camera traps, or genotyping. These studies must have collected both of these sets of data over the same time period and for the same target population. A graphical representation of the filtering process is described using the Preferred Reporting Items for Systematic Reviews and Meta-Analyses chart (Figure 1) [14]. From each paper that passed our filtering criteria, we extracted the focal species, location of study, size of study site, camera data analysis method, and type of genetic sample that was collected (Supplementary Table S1). We also extracted study outcomes including the total number of individuals identified. When necessary, we used the study area size and density estimate to calculate the number of individuals identified. Data depicted only in figures (this happened only once) was extracted using ImageJ (version 1.53i) [15].

We initially compared the number of focal-species individuals identified using live trapping to those made using less invasive methods (camera traps and genetic analyses) using a linear regression. As we were interested in evaluating a 1:1 relationship between the two estimates, we predicted population size using less invasive methods to estimates made using live trapping data, setting the intercept to zero. To meet assumptions of normality, we log-transformed both estimates of population size. We then extracted the residuals for each population from the resulting regression line. We repeated these analyses to compare population size estimates generated from less invasive to each other, where camera trap estimates and genetic data estimates were the predictor and response variables, respectively. We subsequently used both sets of standardized residuals to understand which factors (see below) might be related to the discrepancies between the population size estimates. These and all subsequent statistical analyses were performed in R [16].

We calculated the mean number of individuals identified using trapping compared to less invasive approaches and the mean number of individuals identified using camera traps compared to genetic data analyses using a bootstrapping approach. Specifically, we estimated the mean difference between each group 1000 times, resampling 80% of the values with replacement. We then estimated the 95% confidence interval around the mean difference estimated from the data and compared this interval to zero.

We evaluated the extent to which the differences in number of individuals detected between methods were related to the phylogenetic relatedness of the species in the studies included in our analyses. We used the taxonomic categories of order, family, genus, and species to create a phylogenetic tree of species included in the studies from which we collected population size data, where all branch lengths were set to 1. We then used a phylogenetic least squares regression to quantify the relationship between our phylogeny and the regression residuals described above using a linear model and assuming Brownian evolution [17].

Next, we tested the extent to which properties of each study were related to the differences between number of detected individuals using a series of regressions where standardized residuals from our original regressions were predicted from study characteristics. First, we estimated the effect of camera analysis methods (spatial and random) on the differences between estimates. In the studies we examined, we categorized spatial methods as those that incorporated camera locations and random methods as those that assumed individuals moved randomly with respect to the camera. Next, we quantified the effect of DNA source tissue (hair and scat) used on differences between number of detected individuals in the studies we analyzed. Finally, we considered how study site size contributed to the residual values using a regression where the log of study size, in kilometers, was used to predict the residuals from our initial regressions. For any of the identified comparisons that were significant predictors of the residuals, we calculated the mean number of individuals identified using the two different approaches, again using a bootstrapping approach. As before, we estimated the mean difference between each group 1000 times, resampling 80% of the values with replacement. We then estimated the 95% confidence interval around the mean difference estimated from the data and compared this interval to zero.

In addition to technical study properties, we were also interested in how biological variables of body size and dominant habitat may have contributed to the differences in detected individuals. To test this, we collected mean body size estimates from the list of Mammalian Species Account in the Journal of Mammalogy (by chance, all identified studies in our analysis focused on mammals) and used the log of these values as predictors of the differences between number of detected individuals (i.e., standardized residuals from original regressions). To quantify the effect of habitat, we extracted the dominant habitat ecozone for each population from the Morrone biogeographic realms [18]. We then used these categories as predictors for the standardized residuals.

## 3. Results

We screened a total of 668 studies that were returned from our Web of Science searches and ultimately identified 28 studies that used at least two methods for identifying individuals in a wild landscape (Supplementary Table S1). This included comparisons for 27 populations that compared live trapping to less invasive methods and 17 populations where camera and genetic data were compared. These studies focused on mammals and were conducted in North America, Europe, Asia, and Oceania (Table 1).

**Figure 2:**
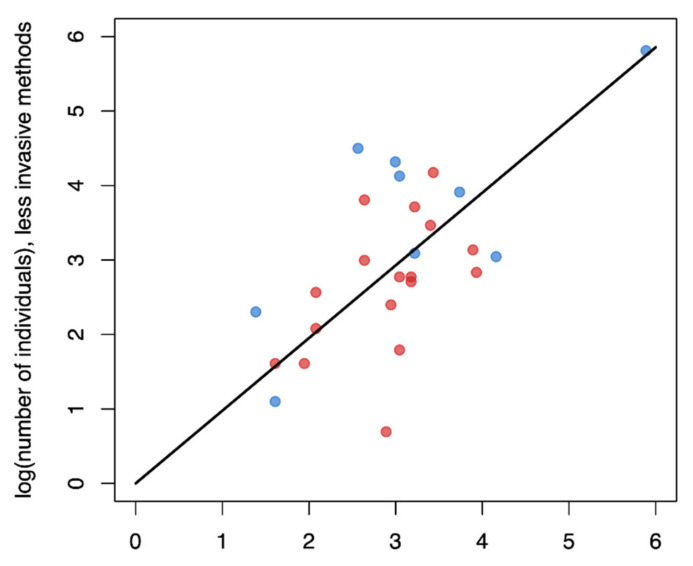
Comparison of the number of individuals identified using live trapping compared to less invasive methods (blue dots denote comparisons where the less invasive method used were camera traps whereas red dots used genetic data). Regression output is depicted by the black line slope = 0.976 ± 0.057, *p* < 0.001, R2 = 0.919, F = 296.1, degrees of freedom = 26). Less invasive methods were on average larger than the population size estimates generated from live trapping efforts (mean = 3.19 individuals).

We were interested in understanding how well minimally invasive approaches matched individual detection data generated from more traditional trapping efforts. Our initial regression, which forced the intercept through zero, suggests that these measures are well correlated with the 95% CIs of slopes overlapping 1 indicating a 1:1 relationship (Table 1; Figure 2). We also quantified the mean difference between these methods and found that minimally invasive methods were similar to population size estimates generated from live trapping efforts (mean difference = 3.19 individuals; 95% CI −8.150 to 15.602).

**Figure 3:**
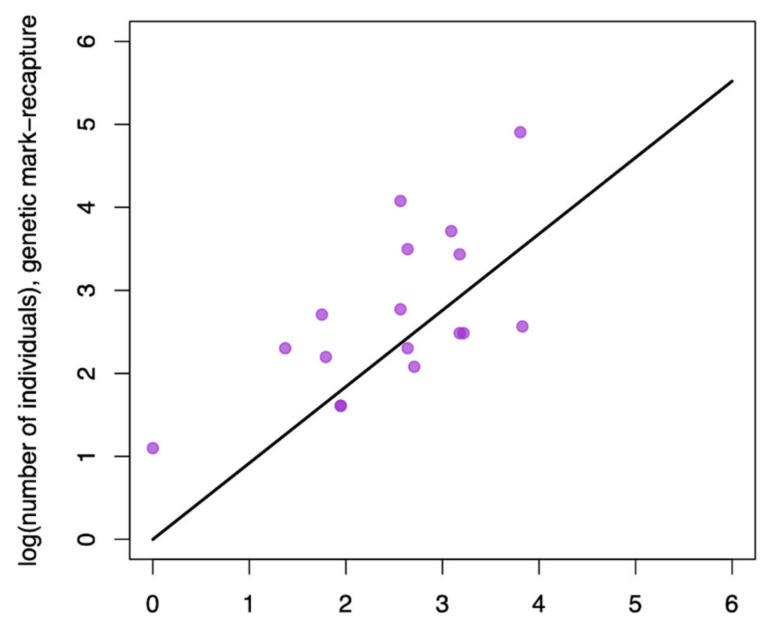
Regression results comparing population size estimates generated using two minimally invasive methods, camera traps and genetic identification of individuals. On average, genetic methods identified an additional 9.07 individuals compared to camera traps (slope = 0.920 ± 0.145, *p* < 0.001, R2 = 0.686, F = 40.29, degrees of freedom = 18).

We also compared identification estimates generated using two minimally invasive methods, camera traps and genetic identification of individuals. Using a regression that forced the intercept through zero, we found that these estimates were reasonably well correlated with the 95% CIs of slopes overlapping 1 (Table 1; Figure 3), even though the explanatory power in this regression was less than the regression comparing trapping to less invasive methods (i.e., R2 = 0.919 compared to R2 = 0.686, respectively; Table 1). We also found that these methods identified a similar number of individuals (genetic methods identified an additional 9.07 individuals compared to camera traps on average; 95% CI −3.323 to 24.212).

As the species in the studies we reviewed were distributed non-randomly across the phylogenetic tree (i.e., all mammals and many species in Carnivora), we considered how the potential confounding variable of shared evolutionary history influenced detection using a phylogenetic least squares regression. We found that phylogeny did not predict the difference in individuals detected using trapping compared to less invasive techniques. Similarly, phylogeny did not predict the difference in detection compared between the two less invasive techniques (Table 1).

We examined our data for evidence of the technical aspects of the study design that may have explained the differences in the number of individuals detected. Although our data were somewhat limited in power, we found that the camera data analysis methodology was a significant predictor of the differences in detection when using the live trapping and camera methods. However, the camera data analysis method did not predict the difference in detection when compared between the less invasive methods (camera vs genetic data analysis). The type of tissue collected for genetic analyses (hair or scat) had no predictive power for detection differences that occurred when using live trapping and genetic methods or when compared between less invasive methods. Finally, we found that study site size did not predict the differences in number of individuals detected in either of our comparisons (Table 1).

Following on the significant relationship between camera data analysis method and regression residuals, we quantified the difference in number of individuals identified using trapping approach and camera data analyzed assuming random movement of individuals or using spatial data information, using bootstrap analysis. We found that when random movement was assumed, trapping approaches identified similar number of individuals compared to camera data (mean = 16 individuals, 95% CI −37.667 to 4.667). When spatial information was incorporated, camera-based approaches identified an average of 35 more individuals compared with trapping-based approaches (95% CI 3.750 to 66.000). However, we note that the number of studies that made these comparisons was small (four and five, respectively) and so these ranges should be interpreted with this limitation in mind.

Finally, we also considered how biological variables influenced the number of individuals detected using the various methods. We found that body size had no predictive power for detection differences compared between live trapping and less invasive methods or when compared between the minimally invasive camera and genetic data methods. We also found no significant difference in predictive power between the biogeographic realms in which these studies were conducted (Table 1).

## 4. Discussion

Overall, the number of individuals detected using trapping and less invasive methods were well correlated. However, on average, 3.19 more individuals were identified using minimally invasive methods than using live trapping and 9.07 more individuals were found with genetic-based estimates compared to camera data-based estimates. Although the boot-strapped confidence intervals around these estimates included zero, these differences may be important in management, particularly in the conservation of endangered species. For example, the California condor is a species of high conservation need that has undergone intensive management [19]. In 1990, less than 50 birds existed in the wild, meaning that an underestimation of close to 9 individuals would represent missing ~18% of the total individuals existing in the wild. These results support the use of minimally invasive methods of trapping, and in particular use of genetic identification-based methods, for quantifying population size particularly when missing a few individuals would substantially undermine conservation or management goals.

In addition to applications to species of extreme conservation concern, minimally invasive approaches may be preferable due to risk mitigation benefits as well as cost and time benefits associated with these methods. Compared to live trapping, less invasive methods offer protections to the focal populations because they are inherently less risky, as animals do not have to be handled. This provides a distinct advantage over live trapping for both animal well-fare and researcher injury risk. In addition, the total cost and effort for less invasive methodologies tends to be less than for invasive or lethal approaches, providing quantitative monetary and time advantages for these methods [20]. Combined with the relative similarity in detection of individuals that occurs with live trapping compared to less invasive methods, the advantages of minimally invasive approaches suggests that these methods should be considered at least as often as live trapping when population monitoring is the goal.

One important limitation of our study was our comparison of the number of individuals detected. Although we would have preferred to compare estimates of population density or other metric that takes into account detection probability, these were not uniformly reported in the studies we identified. However, we suggest that the core ability to identify different numbers of individuals for the same population at the same time provides support for increased sensitivity of minimally invasive methods compared to trapping.

We interpreted the variance around the regression line comparing trapping to less invasive method estimates of population size, as well as the variance around the comparison between minimally invasive methods as a representation of the between-study differences influencing the overall trends. Most of the study parameters we analyzed had no significant relationship on the residuals, however, camera analysis was a significant predictor of the difference in population sizes estimated using live trapping and camera trapping methods. Our analysis supports the idea that including camera trap location yields better individual counts, as evidenced by studies that used these data having identified 35 more individuals (95% CI 3.750 to 66.000) compared with estimates from trapping data, whereas studies that assumed random movement when analyzing camera data identified 16 fewer individuals (95% CI −37.667 to 4.667) compared with trapping-based estimates. However, use of these kinds of models requires consideration of study-specific variables prior to collecting data, as analyzing data not intended for spatial models leads to biased estimates of population size [21].

Although we advocate for the use of some of the less invasive methods available, consideration of species and habitat-specific variables are critical and may require in-field comparisons. For example, method comparisons could be beneficial for species that have low densities and low capture success, such as the southeastern fox squirrel (*Sciurus niger*), whose scarcity and difficulty in being detected requires a reliable method to survey and monitor their populations [22]. Likewise, for species sensitive to habitat loss and fragmentation such as the American marten (*Martes americana*), their density estimates are essential for deciding conservation strategies. American martens positively respond to baited camera traps, suggesting camera traps or baited hair snare traps for genetic analysis may be viable options [23]. However, the decline of marten populations may mean that, at least in some localized areas, genetic diversity is low, meaning that genetic analyses will require additional lab effort to produce individual identification information. Similarly, with Iberian lynx (*Lynx pardinus*) in Spain, where camera trapping is not financially or logistically possible [11], genetic analyses may represent a useful alternative. However, exclusion of wildcats (*Felis sylvestris*) scat, which is similar to Iberian lynx scats, would require extra investments [11]. Therefore, the decision to employ a particular data collection method requires species and ecosystem specific information.

Population estimates of wildlife populations are essential for proper research, conservation, and management. It is integral for the application of conservation and management strategies, such as establishing protections for threatened species, outlining sustainable harvest efforts, and mitigating human-wildlife conflict [24]. The effectiveness of wildlife conservation is heavily dependent on estimates that are accurate and precise to ensure proper decision making, since an inaccurate measurement can lead to a false signal of population stability [24]. Here, we show that minimally invasive methods, including camera and many genetic-based identification methods, detect similar number of individuals compared to trapping-based efforts. Because these methods offer advantages in animal welfare and cost, we suggest increased reliance on minimally invasive methods to generate reliable estimates of population size and density to support on-going management efforts.

## Figures and Tables

**Table 1:** Regression coefficients and standard error (SE) estimates. The first two results describe the comparison of population size estimates generated using two different data collection methods. The remaining results analyzed the ability of several study and species-related predictors to describe the residuals from the initial two regressions. In the table header, F is F statistic, DF is degrees of freedom, and *p* is the *p*-value for the model.

Response Variable	Predictor Variable	Slope Estimate (SE)	Intercept Estimate (SE)	R^2^	F	DF	*p*
Comparison of Population Size Estimates							
live trapping estimates	camera and genetic data estimates	0.976 (0.057)	--	0.919	296.1	26	<0.001
genetic data estimate	camera trapping data estimate	0.920 (0.145)	--	0.686	40.29	18	<0.001
Residuals from Live Trapping vs. Less Invasive Methods							
standardized residuals	phylogenetic tree	--	−0.192 (0.692)	--	--	26	0.783
standardized residuals	camera analysis (spatial or random)	1.547 (0.514)	−0.328 (0.383)	0.502	9.071	8	0.020
standardized residuals	genetic tissue source (hair or scat)	0.191 (0.476)	−0.313 (0.383)	0.000	0.161	16	0.694
standardized residuals	study site size (km^2^)	0.066 (0.084)	0.046 (0.283)	0.000	0.622	23	0.439
standardized residuals	body size (kg)	0.010 (0.141)	0.056 (0.209)	0.000	0.005	25	0.946
standardized residuals	biogeographic realms (Australian, A; nearctic, N; palearctic, P)	--	A:0.102 (0.604) N: −0.146 (0.655) P: 0.233 (0.740)	0.000	0.294	25	0.748
Residuals from Camera vs. Genetic Methods							
standardized residuals	species phylogenetic tree	--	0.332 (0.923)	--	--	18	0.723
standardized residuals	camera data analysis (spatial or random)	0.012 (0.599)	0.433 (0.547)	0.000	0.000	17	0.984
standardized residuals	genetic data tissue source (hair or scat)	0.139 (0.598)	0.327 (0.546)	0.000	0.54	17	0.819
standardized residuals	study site size (km^2^)	−0.822 (0.114)	0.761 (0.494)	0.000	0.519	17	0.481
standardized residuals	body size (kg)	0.114 (0.140)	0.087 (0.486)	0.000	0.673	17	0.424
standardized residuals	biogeographic realms (nearctic, N; neotropical, T; palearctic, P)	--	N: 0.672 (0.337) T: −0.410 (1.010) P: −0.412 (0.463)	0.000	0.416	17	0.667

## Data Availability

All data used in our analyses are available and in Supplementary Materials Table S1. All analysis code available online via GitHub (accessed 30 November 2021): https://github.com/andreamiranda26/Monitoring-Methods-Analysis and https://github.com/jwillou/Monitoring-Methods-Analysis.

## Supplementary Materials

The following are available online at https://www.mdpi.com/article/10.3390/genes12121949/s1, Table S1: Raw data collected from literature and databases used in the analyses in this study.
